# Diagonal Earlobe Crease (Frank’s Sign) for Diagnosis of Coronary Artery Disease: A Systematic Review of Diagnostic Test Accuracy Studies

**DOI:** 10.3390/jcm10132799

**Published:** 2021-06-25

**Authors:** Krzysztof Więckowski, Tomasz Gallina, Andrzej Surdacki, Bernadeta Chyrchel

**Affiliations:** 1 Students’ Scientific Group at Second Department of Cardiology, Institute of Cardiology, Faculty of Medicine, Jagiellonian University Medical College, 2 Jakubowskiego Street, 30-688 Cracow, Poland; tomasz.gallina@student.uj.edu.pl; 2 Second Department of Cardiology, Institute of Cardiology, Faculty of Medicine, Jagiellonian University Medical College, 2 Jakubowskiego, 30-688 Cracow, Poland; andrzej.surdacki@uj.edu.pl (A.S.); bernadeta.chyrchel@uj.edu.pl (B.C.)

**Keywords:** coronary artery disease, coronary syndromes, diagonal earlobe crease, Frank’s sign

## Abstract

Coronary artery disease is a global challenge for healthcare systems. Early diagnosis is a key issue to improve quality of life and reduce morbidity and mortality. Diagonal earlobe crease, a wrinkle extending obliquely across the earlobe, was linked by many authors to various atherosclerotic diseases. This systematic review aimed at summarizing the diagnostic accuracy of diagonal earlobe crease for diagnosis of chronic and acute coronary syndromes in adults. Cochrane’s recommendations for systematic reviews of diagnostic test accuracy studies were followed. The protocol was registered on PROSPERO. Seven electronic databases were searched up to April 2021. The risk of bias and applicability were assessed using the QUADAS-2 tool. Meta-analysis was not performed. Finally, 13 cross-sectional studies evaluating 3951 patients were analyzed, all of which focused on chronic coronary syndromes defined as anatomically significant coronary stenosis. Invasive coronary angiography was used as a reference in most studies, except one which utilized computed tomography angiography. Sensitivity ranged from 26% to 90%, and specificity from 32% to 96%. Positive likelihood ratios varied from 1.11 to 7.03, but most results were below 2. Negative likelihood ratios were from 0.84 to 0.30, but most values exceeded 0.5. Diagnostic accuracy of diagonal earlobe crease for the detection of chronic coronary syndromes is insufficient. It only slightly changes pre-test probability, and its mere presence or absence should not affect the clinical management of the patients. However, for its feasibility and easy interpretation, Frank’s sign could be considered as a part of physical examination.

## 1. Introduction

Coronary artery disease (CAD) is one of the most common cardiovascular disorders and one of the main global causes of death [1]. It usually develops as a result of atherosclerotic obstruction of epicardial coronary arteries [2]. CAD leads to myocardial ischemia and presents as chronic coronary syndromes (CCS) or acute coronary syndromes (ACS) [3]. Early diagnosis is essential to provide an adequate treatment that reduces mortality, eliminates symptoms, and increases the quality of life [4].

**Figure 1:**
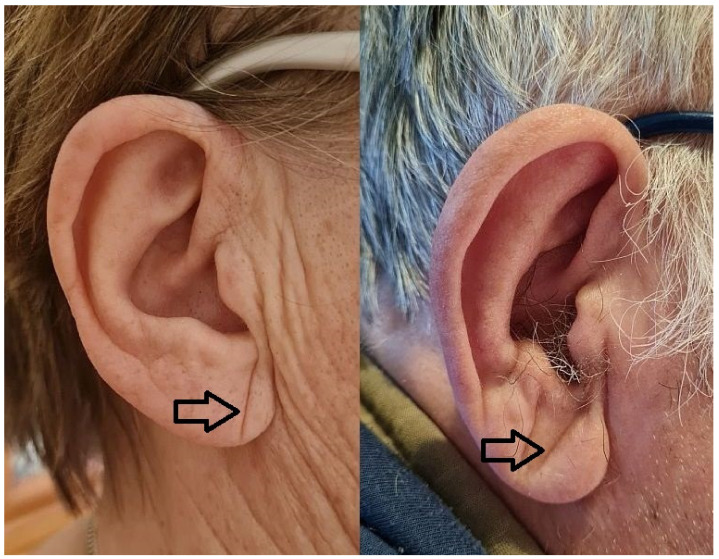
Diagonal earlobe crease in a woman without coronary artery disease and a man with coronary artery disease.

Diagonal earlobe crease (DELC) is a wrinkle extending obliquely from the tragus towards the border of the earlobe, as seen in Figure 1. It was firstly described by Frank in 1973 in the *New England Journal of Medicine* in a case series of patients with CAD [5]. Since then, there have been many reports published regarding its association mainly with atherosclerosis, especially CAD [6]. However, it is barely known and rarely used, likely because its clinical application has not been well established. Noteworthily, the examination of DELC is effortless, non-invasive, and easy to interpret. It could be used in primary care or emergency departments if its diagnostic accuracy is sufficient to support decision-making.

In 2020, Stoyanov et al. published research investigating earlobes along with cardiac samples in the autopsy study [7]. Histopathological examination of DELC-positive earlobes showed myoelastofibrosis in the arterial vessel located at the base of the earlobe, fibrosis, and Wallerian-like degeneration with eosinophilic inclusions in the peripheral nerves. The authors stated that this location is a line of merging of preformed structures prenatally, and thus it may be susceptible to chronic hypoxia–reoxygenation injury due to atherosclerosis. Moreover, they revealed increased cardiac weight and left and right ventricular thickness in DELC-positive patients, and no difference in age between groups. This study supports the hypothesis that DELC is not a random finding but is directly linked to atherosclerosis. Previous studies on the pathophysiology of DELC raised some other mechanisms of its formation, including skin aging [8], collagen degeneration [9], or telomere shortening [10].

We aimed at summarizing the diagnostic accuracy of the DELC for the diagnosis of CCS and ACS in adults.

## 2. Materials and Methods

This systematic review was reported according to the Preferred Reporting Items for Systematic Review and Meta-Analysis [11]. Cochrane’s recommendations for systematic reviews of diagnostic accuracy studies were followed [12]. The protocol was registered on PROSPERO (CRD42021229551).

We focused on two adult populations—patients with suspected CCS referred to further cardiologic evaluation, and patients with suspected ACS who presented in the emergency department. DELC was defined as a wrinkle extending obliquely from the tragus towards the border of the earlobe, and studies that evaluated DELC as an index test were included in the analysis. Reference standards had to agree with the present recommendations of The European Society of Cardiology [3,13] for diagnosis of CCS and ACS. Cross-sectional studies were considered for inclusion, whereas case-control ones were excluded because of the evaluation of nonrepresentative populations due to separate sampling of patients and overestimation of diagnostic accuracy [12]. Only full-text publications were accepted.

Seven electronic databases, including Pubmed MEDLINE, Embase, Web of Science, Cochrane CENTRAL, CINAHL, Clinicaltrials.gov, and Scopus, were searched up to 8 January 2021 without any restrictions on the language or date of publication. The search included the terms “Frank’s sign” and “earlobe crease” with variants. The full search strategy is reported in Supplementary Materials File S1. Additionally, we manually screened the references of all papers regarding DELC. The search was repeated on 13 April 2021, and no new eligible papers were found.

All titles and abstracts were screened, and potentially eligible papers were chosen for full-text assessment, which resulted in the inclusion of studies according to the criteria mentioned above. All relevant data, including study design, population, index test, reference standard characteristics, and study results were extracted. The Quality Assessment of Diagnostic Accuracy Studies 2 (QUADAS-2) tool was used to assess the methodological quality [14]. Every stage was preceded by a calibration round to ensure understanding of the criteria and was performed by two reviewers (K.W., T.G.) independently. Any disagreements were resolved by discussion and help from the third reviewer (A.S.) if necessary.

Diagnostic accuracy of each study presented as sensitivity, specificity, and positive and negative likelihood ratios (LR+ and LR−) with 95% confidence intervals (95% CI) calculated from true positive (TP), false positive (FP), false negative (FN), and true negative (TN) values. Analyses were carried out in Review Manager Version 5.4. (Copenhagen, The Nordic Cochrane Centre, The Cochrane Collaboration, 2020) and OriginPro Version 2021 (OriginLab Corporation, Northampton, MA, USA).

Separate analyses of diagnostic accuracy depending on some variables, including sex, age, location, DELC definition, reference standards, and methodological quality were planned to be done if relevant data were available. Due to the preliminary search as well as analysis of previous reviews, we expected considerable heterogeneity between studies, and the decision was made not to perform a meta-analysis.

## 3. Results

**Figure 2:**
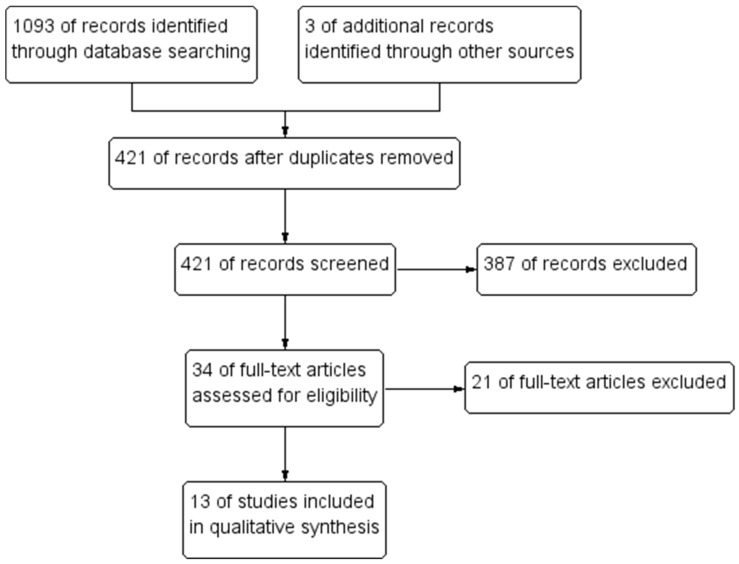
Flowchart of studies selection.

The study selection flowchart is presented in Figure 2. From the 421 unique records identified through database search, 34 full texts were assessed, and 13 studies were finally included.

Reasons for exclusion of full texts were as follows: not relevant population [9], reference standard [15,16,17,18,19,20,21], target condition [22], and case-control design [23,24,25]. Many probable errors in methodology description and data presentation were present in the research by Bawaskar et al., making it not possible to interpret [26]. Two records were letters [27,28] and one was a subgroup analysis from another study included in our analysis [29,30]. Five studies published in the years 1979–1990 were not accessible [31,32,33,34,35]. Detailed explanations are presented in Supplementary Materials File S3.

Characteristics of included studies are detailed in Table 1. The total number of patients was 3,951. Six studies were conducted in North America, five in Asia, one in Europe, and one in South America. Most of them were carried out on both sexes, whereas three included only men. Various descriptions of the age of the population were used, and available data are presented in Table 1. The total prevalence of DELC was 60.5% and varied from 17.0% to 73.0%. The overall prevalence of CAD was 53.9% and ranged from 16.5% to 80.8%.

Generally, most studies defined DELC as at least a unilateral sign; in two papers bilateral DELC was required to be considered as a positive, and another two presented results for both definitions. Some studies showed no more than information of diagonal creasing of earlobe without any other details [8,36]. In another study, only the word “significant” was added to the positive sign description [37]. One accepted any crease [38], whereas others provided detailed thresholds of the relation of the crease to the whole length of the earlobe, which varied from more than one-third [39], at least half [40,41], at least two-thirds [42,43], or 100% [29,44,45,46]. The combination of these approaches leads to the finding that DELC was defined in nine different ways.

All studies evaluated the diagnostic accuracy of DELC in the diagnosis of CCS. The reference standard was invasive coronary angiography (ICA), except one that utilized computed tomography angiography. In both, the threshold for anatomically significant stenosis was 50%.

**Figure 3:**
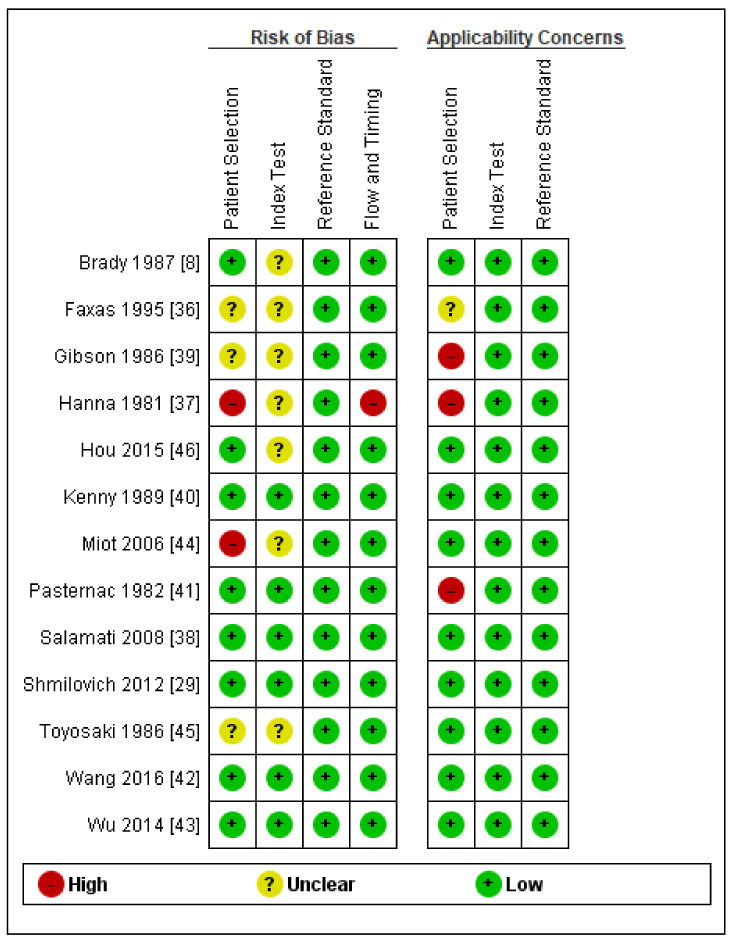
Results of quality assessment of individual studies.

**Figure 4:**
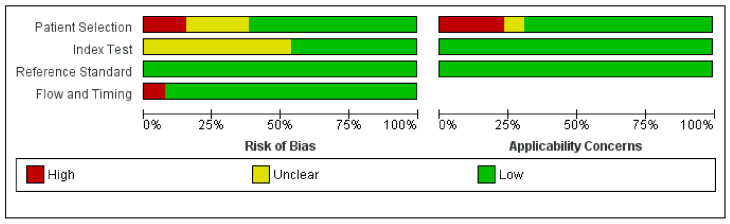
Summarized results of quality assessment.

Results of quality assessment using the QUADAS-2 tool are presented in Figure 3 and Figure 4. Detailed explanations of our decisions are provided in Supplementary Materials File S2. Only five were judged as having a low risk of bias and concern of applicability in all domains. The most important limitation was patient selection because of unclear indications for performing ICA or inclusion of some patients that did not precisely match the review question. The main issues, which resulted in reduced ratings in the index test domains, were the unclear pre-specified definition of DELC and unclear blinding of the results of the reference standards. Reference standard domains were rated as having the highest quality. One study performed ICA and final analysis only on the subgroup of the total number of patients, which resulted in a high risk of bias in the Flow and Timing domain.

**Figure 5:**
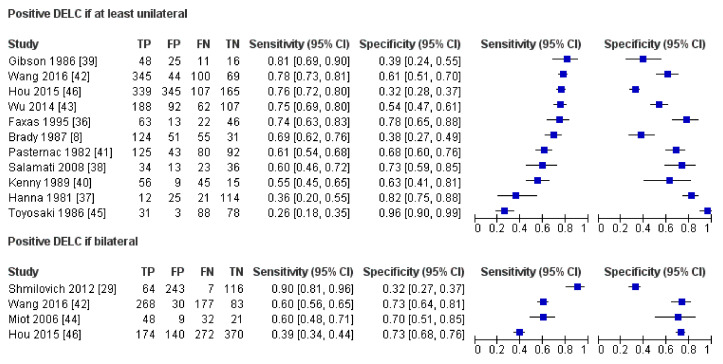
Results of individual studies and forest plot of sensitivity and specificity with 95% confidence intervals.

**Figure 6:**
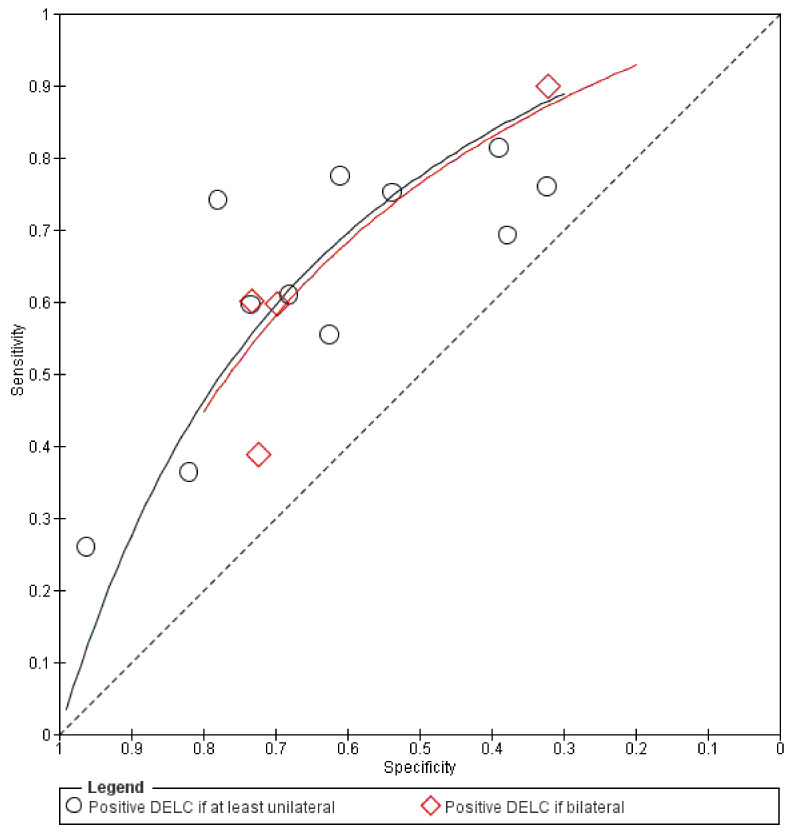
Summary receiver operating characteristic plot.

**Figure 7:**
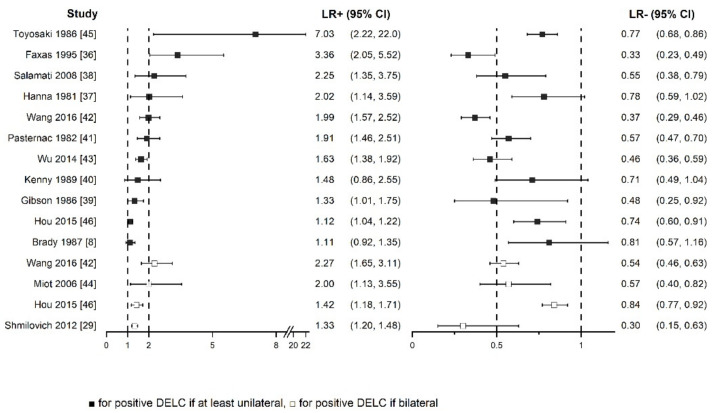
Forest plot of positive and negative likelihood ratios with 95% confidence intervals.

**Figure 8:**
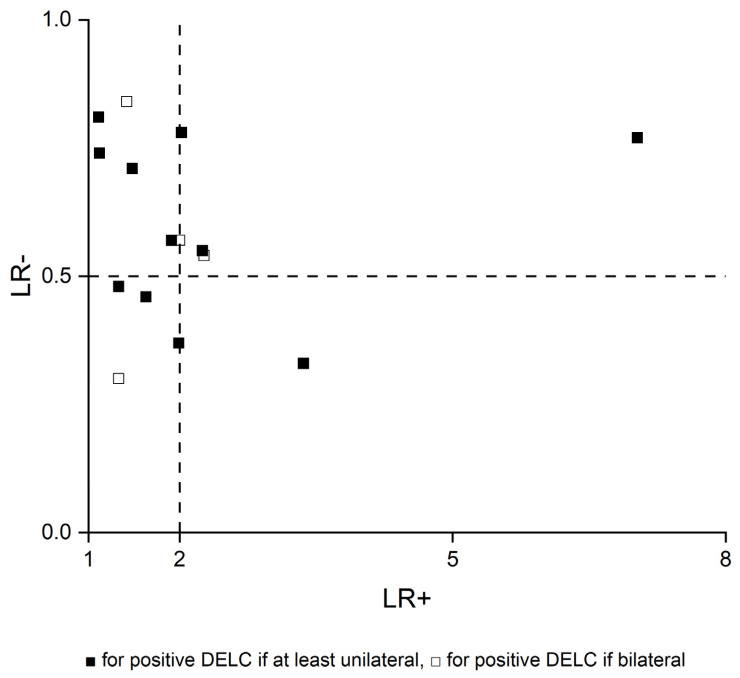
Scatter plot of positive and negative likelihood ratios.

Results of all included studies are detailed in Figure 5, Figure 6, Figure 7 and Figure 8. TP, FP, FN, and TN values of each study with calculated sensitivity and specificity with 95% CIs are presented in Figure 5. Sensitivity and specificity were also summarized by the summary receiver operating characteristic plot in Figure 6. LR+ and LR− with 95% CI are presented in Figure 7 and are summarized in Figure 8.

Due to the considerable heterogeneity, especially in the DELC definition, and the lack of raw data in many studies necessary to calculate diagnostic accuracy for subgroups of different sex and age, we decided not to perform additional analyses. In our opinion, such analyses of the mentioned subgroups as well as different locations or methodological quality would not change the final appraisal of the usefulness of DELC. Apart from that, we presented separately the results of studies that considered positive DELC when at least unilateral, and these where only bilateral DELC was accepted. Meta-analysis was not performed in this systematic review.

## 4. Discussion

This systematic review aimed at summarizing the diagnostic accuracy of diagonal earlobe crease for the diagnosis of chronic and acute coronary syndromes. Finally, only studies evaluating CCS with anatomically significant stenosis of coronary arteries detected mostly in invasive coronary angiography, except one which utilized computed coronary angiography, were eligible. During the diagnostic process of CCS, the pre-test probability of obstructive CAD is assessed basing on medical history, physical examination, and additional tests to choose an appropriate final invasive or non-invasive test. Since ICA is offered to patients with a high clinical likelihood of disease, the results of this systematic review should be mainly referred to this population and carefully generalized to other patients [3].

Sensitivity and specificity showed notable heterogeneity, whereas calculated LRs+ and LRs− were more consistent. Generally, LR+ above 2 and LR− less than 0.5 are values that increase or decrease pre-test probability approximately by 15 percentage points or more. These are the most used cut-off points, which set the threshold of informativeness of the diagnostic tests. However, values in the range from 2 to 5 and from 0.5 to 0.2, respectively, provide only a small change in probability [47]. In most studies in this systematic review, LR+ and LR− were even below 2 and above 0.5, respectively. Basing on the collected data, we suppose that DELC has insufficient diagnostic value to change the clinical management of patients. However, as its examination could slightly change CAD probability, it could be considered as a part of the physical examination of patients with suspected CAD.

A systematic review and meta-analysis performed by Knuuti et al. investigated the diagnostic accuracy of non-invasive tests in the detection of significant coronary stenosis [48]. Thirteen studies with 2442 patients focused on stress electrocardiography. Pooled sensitivity, specificity, and LR+ and LR− with 95% CI were as follows: 58% (46–69%), 62% (54–69%), 1.53 (1.21–1.94), and 0.68 (0.49–0.93). Thus, the authors stated that the practical utility of stress electrocardiography in this area is limited, but they also emphasized other useful information, such as exercise tolerance or arrhythmias. Even though meta-analysis was not performed in our systematic review, we conclude that the diagnostic accuracy of DELC is comparable to the mentioned measures of stress electrocardiography in detecting anatomically significant coronary stenosis.

We have found two other systematic reviews which aimed at evaluating the diagnostic performance of DELC in this matter.

Lucenteforte et al. published a systematic review of 37 studies, of which five were also enrolled in our review [49]. The study did not define a specific research question that would cover all components of the patient, index test, comparison, outcome, and study type (PICOS) framework. Among included studies there are 17 case-control studies which mostly assigned patients to cases or controls based only on patients’ medical histories. In only a few cases was angiographically verified. Moreover, even three autopsy research studies were evaluated, which are detached from real-life clinical scenarios. Five records were short letters to editors. Additionally, various thresholds for anatomically significant stenosis—50%, 70% or 75%—were accepted. In one study, target condition was defined as cardiovascular disease and was combined with coronary artery disease, cerebrovascular disease, and others. Despite notable differences between studies in all areas, results were pooled in a meta-analysis. Furthermore, methods were not established in advance in the protocol, the extensive search strategy was not applied as authors had searched only the MEDLINE database, and quality assessment of the included studies was not done.

At the moment, the systematic review by Curtis et al. is available at Authorea Preprint Repository [50]. The authors aimed at including only studies where presence or absence of DELC was compared to the diagnosis of CAD made using ICA. Of 12 included studies there were six that were also enrolled in our systematic review. However, four others were case-controlled, where only cases had angiographically confirmed disease and control groups comprised asymptomatic patients without known CAD. One study was a short letter to the editor. Similarly, various thresholds for anatomically significant stenosis—mostly 50%, but in three studies 70% or 75%—were accepted. One research study was assessed by us in the full-text stage but was excluded due to a not relevant population, as it evaluated mostly invited patients who had undergone ICA in previous years. Despite considerable heterogeneity between studies, especially in DELC definition as highlighted in our review, diagnostic odds ratios were pooled in a meta-analysis. Additionally, the probable reason of not including other potentially eligible studies identified by us is that it was stated that only the MEDLINE database was searched.

Authors of both mentioned systematic reviews concluded that DELC could be considered as a marker of CAD. We generally agree with their findings, but we are far more cautious in recommending routine examination of this sign. DELC could be a part of a physical examination only along with thorough clinical assessment. Moreover, it must be highlighted that available evidence comes mostly from populations of patients with relatively high pre-test probability of CAD, and generalizability of these results is uncertain. Additionally, the reference standard was mainly invasive coronary angiography, which has its own limitations as well [51].

We would like to emphasize that DELC could hypothetically be used in other clinical scenarios. For instance, it may be a part of screening for CCS in asymptomatic adults or could be used to estimate the risk of myocardial infarction in the general population.

Limitations of this systematic review are mainly related to considerable heterogeneity between included studies that have been published over the last 40 years and were conducted on four continents. Additionally, various DELC definitions were used. In a few studies, the prevalence of DELC or CAD stood out from other studies. Second, this systematic review finally focused only on chronic coronary syndromes.

## 5. Conclusions

In conclusion, in patients with a high clinical likelihood of obstructive coronary artery disease, diagonal earlobe crease only slightly changes pre-test disease probability, which implies that its diagnostic accuracy in the detection of anatomically significant coronary stenosis is insufficient. Although the mere presence or absence of diagonal earlobe should not affect the clinical management, it could be considered as a part of physical examination for its feasibility and easy interpretation, Nevertheless, further research is necessary to better establish the diagnostic performance of Frank’s sign or its other potential clinical applications.

## Figures and Tables

**Table 1:** Characteristics of included studies.

Author (Year)	Country	Sample Size (n)	Males	Age ^1^	DELC Prevalence	CAD Prevalence	DELC Definition	Reference Standard
Brady (1987) [8]	USA	261	100%	DELC+ 60	67%	69%	At least unilateral	ICA
				DELC− 52				
Gibson (1986) [39]	USA	100	68%	Males 65	73%	59%	At least unilateral	ICA
				Females 70				
Faxas (1995) [36]	Cuba	144	NA	NA	53%	59%	At least unilateral	ICA
Hanna (1981) [37]	USA	172	100%	94% below 50	22%	19%	At least unilateral	ICA
Hou (2015) [46]	China	956	57%	CAD+ 55 ± 9	72%, 33%	47%	At least unilateral, bilateral	ICA
				CAD− 51 ± 8				
Kenny (1989) [40]	Ireland	125	90%	Range 35–90	52%	81%	At least unilateral	ICA
Miot (2006) [44]	Brazil	110	100%	58 ± 12	52%	73%	Bilateral	ICA
Pasternac (1982) [41]	Canada	340	74%	50 ± 8	49%	60%	At least unilateral	ICA
Salamati (2008) [38]	Iran	106	66%	50 ± 14	44%	54%	At least unilateral	ICA
Shmilovich (2012) [29]	USA	430	61%	61 ± 13	71%	17%	Bilateral	CTA
Toyosaki (1986) [45]	Japan	200	76%	72% above 50	17%	60%	At least unilateral	ICA
Wang (2016) [42]	China	558	72%	64	70%, 53%	80%	At least unilateral, bilateral	ICA
Wu (2014) [43]	China	449	62%	63 ± 12	62%	56%	At least unilateral	ICA

^1^ Mean age ± standard deviation or other if specified; CAD, coronary artery disease; CTA, computed tomography angiography; DELC, diagonal earlobe crease; ICA, invasive coronary angiography; NA, not available.

## Supplementary Materials

The following are available online at https://www.mdpi.com/article/10.3390/jcm10132799/s1, File S1: Full search strategy, File S2: Detailed quality assessment using QUADAS-2, File S3: List of 21 excluded studies at full-text assessment stage with reasons.
