# Supplementary material for: Diagonal Earlobe Crease (Frank’s Sign) for Diagnosis of Coronary Artery Disease: A Systematic Review of Diagnostic Test Accuracy Studies
Source: J Clin Med. 2021 Jun 25;10(13):2799. doi: 10.3390/jcm10132799 (PMC8268092; doi:10.3390/jcm10132799)
Supplement: Supplementary file 1 [file jcm-10-02799-s001.zip › File S1.pdf]

Detailed search strategy

Pubmed MEDLINE [1]

"Frank's sign"[Title/Abstract] OR "Frank sign"[Title/Abstract] OR "Franks sign"[Title/Abstract] OR "earlobe* crease*"[Title/Abstract] OR "ear lobe* crease*"[Title/Abstract] OR "ear-lobe* crease*"[Title/Abstract] OR "ear* crease*"[Title/Abstract]

Embase [2]

'frank/s sign':ti,ab,kw OR 'frank$ sign':ti,ab,kw OR 'earlobe$ crease$':ti,ab,kw OR 'ear lobe$ crease$':ti,ab,kw OR 'ear-lobe$ crease$':ti,ab,kw OR 'ear$ crease$':ti,ab,kw OR 'earlobe crease'/exp OR 'frank sign'/exp

Web of Science [3]

TS=("Frank's sign" OR "Frank$ sign" OR “earlobe* crease$” OR “ear lobe* crease$” OR “ear-lobe* crease$” OR “ear* crease$”)

The Cochrane Central Register of Controlled Trials (CENTRAL) [4]

"Frank's sign" OR "Frank sign" OR "Franks sign" OR "earlobe crease" OR "earlobe creases" OR "earlobes crease" OR "earlobes creases" OR "ear lobe crease" OR "ear lobe creases" OR "ear lobes crease" OR "ear lobes creases" OR "ear-lobe crease" OR "ear-lobe creases" OR "ear-lobes crease" OR "ear-lobes creases" OR "ear crease" OR "ear creases" OR "ears crease" OR "ears creases"

CINAHL [5]

"Frank's sign" OR "Frank# sign" OR “earlobe# crease#” OR “ear lobe# crease#” OR “ear-lobe# crease#” OR “ear# crease#”

Clinicaltrials.gov [6]

"Frank's sign" OR "Frank sign" OR "Franks sign" OR "earlobe crease" OR "earlobe creases" OR "earlobes crease" OR "earlobes creases" OR "ear lobe crease" OR "ear lobe creases" OR "ear lobes crease" OR "ear lobes creases" OR "ear-lobe

crease" OR "ear-lobe creases" OR "ear-lobes crease" OR "ear-lobes creases" OR "ear crease" OR "ear creases" OR "ears crease" OR "ears creases"

Scopus [7]

"Frank's sign" OR "Frank* sign" OR “earlobe* crease*” OR “ear lobe* crease*” OR “ear-lobe* crease*” OR “ear* crease*”

References

1. Pubmed MEDLINE. Available online: https://pubmed.ncbi.nlm.nih.gov/ (accessed on 8 January 2021)

2. Embase. Available online: https://www.embase.com/ (accessed on 8 January 2021)

3. Web of Science. Available online: https://webofknowledge.com/

4. CENTRAL. Available online: https://www.cochranelibrary.com/central (accessed on 8 January 2021)

5. CINAHL. Available online: https://www.ebsco.com/products/research-databases/cinahl-database (accessed on 8 January 2021)

6. ClinicalTrials.gov. Available online: https://clinicaltrials.gov/ (accessed on 8 January 2021)

7. Scopus. Available online: https://www.scopus.com/ (accessed on 8 January 2021)
