# Supplementary material for: Diagonal Earlobe Crease (Frank’s Sign) for Diagnosis of Coronary Artery Disease: A Systematic Review of Diagnostic Test Accuracy Studies
Source: J Clin Med. 2021 Jun 25;10(13):2799. doi: 10.3390/jcm10132799 (PMC8268092; doi:10.3390/jcm10132799)
Supplement: Supplementary file 1 [file jcm-10-02799-s001.zip › File S2.pdf]

Detailed quality assessment using The Quality Assessment of Diagnostic Accuracy Studies 2 (QUADAS-2) tool. Numbering of references are in line with the main body of the article.

**First author (year)**	**Brady (1987) [8]**				
**Population**	„261 men who had been admitted to the University of Massachusetts Medical Center, Worcester, for coronary arteriography”				
**Index test**	Positive if „either a unilateral or bilateral ELC”, not precisely defined				
**Reference standard and target condition**	„Classification of coronary artery disease at coronary arteriography included normal coronary arteries (no lesions detected), minor lesions (<50% occlusion), and single-vessel (>50% occluded), double-vessel, and triple-vessel disease.”				
**QUADAS-2 domain**		QUADAS-2 item	Description	Judgement	
**Patient selection**	Risk of bias	Consecutive or random sample enrolled?	„consecutive”	Yes	Low
		Case-control design avoided?	Cross-sectional design	Yes	
		Inappropriate exclusions avoided?	No one excluded from 261 consecutive patients	Yes	
	Concerns regarding applicability		Indications for coronary angiography not stated – however it was a group of consecutive patients who underwent planned procedure		Low
**Index test**	Risk of bias	Interpreted without knowledge of the results of the reference standard?	„assessed prior to the catheterization”	Yes	Unclear
		Pre-specified threshold?	Not precisely defined	Unclear	
	Concerns regarding applicability		In line with the review question		Low
**Reference standard**	Risk of bias	Likely to correctly classify the target condition?	„Classification of coronary artery disease at coronary arteriography (...) vessel (>50% occluded)”	Yes	Low
		Interpreted without knowledge of the results of the index test?	Not stated	Unclear	
	Concerns regarding applicability		In line with the review question		Low
**Flow and timing**	Risk of bias	Appropriate interval between index test and reference standard?	Both examined during one visit	Yes	Low
		All patients received the same reference standard?	Coronary angiography performed on all 261 patients	Yes	

		All patients included in the analysis?	Results of all 261 patients presented	Yes	

**First author (year)**	**Faxas (1995) [36]**				
**Population**	144 patients undergoing coronary angiography				
**Index test**	Earlobe crease positive if at least unilateral, not precisely defined				
**Reference standard and target condition**	Lesions of coronary arteries in coronary angiography were divided into not significant if less than 50% occluded and significant if more than 50% stenosis present				
**QUADAS-2 domain**		QUADAS-2 item	Description	Judgement	
**Patient selection**	Risk of bias	Consecutive or random sample enrolled?	Not stated	Unclear	Unclear
		Case-control design avoided?	Cross-sectional design	Yes	
		Inappropriate exclusions avoided?	Not stated	Unclear	
	Concerns regarding applicability		Patients undergoing coronary angiography regardless of indication		Unclear
**Index test**	Risk of bias	Interpreted without knowledge of the results of the reference standard?	Not stated	Unclear	Unclear
		Pre-specified threshold?	Not stated	Unclear	
	Concerns regarding applicability		In line with the review question		Low
**Reference standard**	Risk of bias	Likely to correctly classify the target condition?	More than 50% stenosis in coronary angiography	Yes	Low
		Interpreted without knowledge of the results of the index test?	Not stated	Unclear	
	Concerns regarding applicability		In line with the review question		Low
**Flow and timing**	Risk of bias	Appropriate interval between index test and reference standard?	Both examined during one visit	Yes	Low
		All patients received the same reference standard?	Coronary angiography performed on all 144 patients	Yes	
		All patients included in the analysis?	Results of all 144 patients presented	Yes	

First author (year)	Gibson (1986) [39]				
**Population**	„100 patients with symptomatic aortic stenosis undergoing cardiac catheterization to establish the hemodynamic severity of the obstruction and the degree of coronary artery involvement”				
**Index test**	Positive if „deep indentation connecting the tragus to the lobe edge” or more than „superficial crease” and measuring more than a third of the distance from the tragus to the earlobe edge, at least unilateral				
**Reference standard and target condition**	„Significant coronary artery disease was defined as narrowing ≥50%” in „coronary arteriograms”				
**QUADAS-2 domain**		QUADAS-2 item	Description	Judgement	
**Patient selection**	Risk of bias	Consecutive or random sample enrolled?	Not stated	Unclear	Unclear
		Case-control design avoided?	Cross-sectional design	Yes	
		Inappropriate exclusions avoided?	Not stated	Unclear	
	Concerns regarding applicability		„patients with symptomatic aortic stenosis”		High
**Index test**	Risk of bias	Interpreted without knowledge of the results of the reference standard?	„On the day of cardiac catheterization patients were examined for the presence or absence of ear lobe creases”	Unclear	Unclear
		Pre-specified threshold?	Definition above	Yes	
	Concerns regarding applicability		In line with the review question		Low
**Reference standard**	Risk of bias	Likely to correctly classify the target condition?	„Significant coronary artery disease was defined as narrowing ≥50%”	Yes	Low
		Interpreted without knowledge of the results of the index test?	Not stated	Unclear	
	Concerns regarding applicability		In line with the review question		Low
**Flow and timing**	Risk of bias	Appropriate interval between index test and reference standard?	„On the day of cardiac catheterization patients were examined for the presence or absence of ear lobe creases”	Yes	Low
		All patients received the same reference standard?	Coronary angiography performed on all 100 patients	Yes	
		All patients included in the analysis?	Results of all 100 patients presented	Yes	

First author (year)	Hanna (1981) [37]				
**Population**	„apparently healthy USAF flyers who were referred for medical evaluation because of a finding such as nonspecific T wave changes on a routine ECG or because of an event, such as syncope, that raised the question of medical qualification for flying duties”				
**Index test**	Positive if „significant crease”, at least unilateral				
**Reference standard and target condition**	„Coronary angiograms were classified into three categories: normal (judged to be completely free of any luminal obstruction or intimal roughening), <50% (evidence of luminal obstruction(s) with the maximum diameter narrowing less than 50%), and ≥50% (at least one lesion with a 50% or greater obstruction).”				
**QUADAS-2 domain**		QUADAS-2 item	Description	Judgement	
**Patient selection**	Risk of bias	Consecutive or random sample enrolled?	Patients selected for coronary angiography	No	High
		Case-control design avoided?	Cross-sectional	Yes	
		Inappropriate exclusions avoided?	Not stated	Unclear	
	Concerns regarding applicability		„apparently healthy USAF flyers”		High
**Index test**	Risk of bias	Interpreted without knowledge of the results of the reference standard?	„had no knowledge of the angiographic findings at the time the photographs were scored for the presence or absence of earlobe crease”	Yes	Unclear
		Pre-specified threshold?	Unclear definition	Unclear	
	Concerns regarding applicability		In line with the review question		Low
**Reference standard**	Risk of bias	Likely to correctly classify the target condition?	„Coronary angiograms were classified into (...) ≥50% (at least one lesion with a 50% or greater obstruction).”	Yes	Low
		Interpreted without knowledge of the results of the index test?	„The coronary angiograms were interpreted independently by (...) cardiologists who were not involved in this study”	Yes	
	Concerns regarding applicability		In line with the review question		Low
**Flow and timing**	Risk of bias	Appropriate interval between index test and reference standard?	Both examined during one visit	Yes	High
		All patients received the same reference standard?	Coronary angiography performed on only 172 from 1172 patients	No	

		All patients included in the analysis?	Results of 172 patients who underwent coronary angiography presented	No	

First author (year)	Hou (2015) [46]				
**Population**	„956 consecutive patients undergoing angiography in our hospital”				
**Index test**	Positive if „wrinkle-like line extending diagonally from the tragus across the lobule to the rear edge of the auricle of the ear”, results for unilateral and bilateral sign presented				
**Reference standard and target condition**	„Luminal stenosis was defined as significant when the luminal diameter of the major coronary artery, or one of its major branches, showed a reduction of ≥50%”				
**QUADAS-2 domain**		QUADAS-2 item	Description	Judgement	
**Patient selection**	Risk of bias	Consecutive or random sample enrolled?	„consecutive”	Yes	Low
		Case-control design avoided?	Cross-sectional design	Yes	
		Inappropriate exclusions avoided?	„Subjects with external ear disease or deformation, ear injury history, skin disease affecting ears, previous diagnosis of CAD or ischemic stroke, myocardial infarction, cardiomyopathy, and chronic diseases, such as cancer, were excluded”	Yes	
	Concerns regarding applicability		Indications for coronary angiography not stated however it was a group of consecutive patients and we assume that substantial percent underwent planned procedure		Low
**Index test**	Risk of bias	Interpreted without knowledge of the results of the reference standard?	Not stated	Unclear	Unclear
		Pre-specified threshold?	Definition above	Yes	
	Concerns regarding applicability		In line with the review question		Low
**Reference standard**	Risk of bias	Likely to correctly classify the target condition?	„Luminal stenosis (...) showed a reduction of ≥50%”	Yes	Low

		Interpreted without knowledge of the results of the index test?	Not stated	Unclear	
	Concerns regarding applicability		In line with the review question		Low
**Flow and timing**	Risk of bias	Appropriate interval between index test and reference standard?	Both examined during one visit	Yes	Low
		All patients received the same reference standard?	Coronary angiography performed on all 956 patients	Yes	
		All patients included in the analysis?	Results of all 956 patients presented	Yes	

**First author (year)**	**Kenny (1989) [40]**				
**Population**	„125 consecutive patients undergoing coronary angiography”				
**Index test**	„An ear lobe crease was judged present if it creased the skin to a depth of ≥1 mm and measured, to the nearest millimeter, 50% or greater the diameter of the ear lobe”, positive if at least unilateral				
**Reference standard and target condition**	„50% stenosis was chosen at this was considered significant” in coronary angiography				
**QUADAS-2 domain**		QUADAS-2 item	Description	Judgement	
**Patient selection**	Risk of bias	Consecutive or random sample enrolled?	„consecutive”	Yes	Low
		Case-control design avoided?	Cross-sectional design	Yes	
		Inappropriate exclusions avoided?	„only females who wore earrings were excluded”	Yes	
	Concerns regarding applicability		Indications for coronary angiography not stated however it was a group of consecutive patients who underwent planned procedure		Low
**Index test**	Risk of bias	Interpreted without knowledge of the results of the reference standard?	„examined (...) on the afternoon of admission to the hospital”	Yes	Low
		Pre-specified threshold?	Definition above	Yes	
	Concerns regarding applicability		In line with the review question		Low
**Reference standard**	Risk of bias	Likely to correctly classify the target condition?	„50% stenosis was chosen at this was considered significant”	Yes	Low

		Interpreted without knowledge of the results of the index test?	Not stated	Unclear	
	Concerns regarding applicability		In line with the review question		Low
**Flow and timing**	Risk of bias	Appropriate interval between index test and reference standard?	Both examined during one visit	Yes	Low
		All patients received the same reference standard?	„All patients underwent coronary arteriography"	Yes	
		All patients included in the analysis?	Results of all 125 patients presented	Yes	

**First author (year)**	**Miot (2006) [44]**				
**Population**	„110 male adults who had been submited to coronary angiography"				
**Index test**	Positivie if „bilateral creases and those affecting the total whole extension of the lobe"				
**Reference standard and target condition**	„with greater than 50% obstruction of at least one coronary artery"				
**QUADAS-2 domain**		QUADAS-2 item	Description	Judgement	
**Patient selection**	Risk of bias	Consecutive or random sample enrolled?	Not stated	Unclear	High
		Case-control design avoided?	„case-control", however coronary angiography was done in all controls and authors stated that „The 2.5:1 ratio between cases and controls was determined to reduce the possibility of random error, considering the difficulty of having adult patients with normal exams"	Unclear	
		Inappropriate exclusions avoided?	Study design detailed above led to exclusion of some unknown patients	No	
	Concerns regarding applicability		Indications for coronary angiography not stated however it was a group of consecutive patients and we assume that substantial percent underwent planned procedure		Low

**Index test**	Risk of bias	Interpreted without knowledge of the results of the reference standard?	Not stated	Unclear	Unclear
		Pre-specified threshold?	Definition above	Yes	
	Concerns regarding applicability		In line with the review question		Low
**Reference standard**	Risk of bias	Likely to correctly classify the target condition?	„with greater than 50% obstruction of at least one coronary artery”	Yes	Low
		Interpreted without knowledge of the results of the index test?	Not stated	Unclear	
	Concerns regarding applicability		In line with the review question		Low
**Flow and timing**	Risk of bias	Appropriate interval between index test and reference standard?	Both examined during one visit	Yes	Low
		All patients received the same reference standard?	Coronary angiography performed on all 110 patients	Yes	
		All patients included in the analysis?	Results of all 110 patients presented	Yes	

**First author (year)**	**Pasternac (1982) [41]**				
**Population**	„340 patients consecutively admitted to the Montreal Heart Institute for coronary arteriography”				
**Index test**	„examiner assessed the earlobe for the presence of a clear-cut diagonal crease of the lobular portion of either auricle”, „in whom the crease extended for a distance greater than half the diagonal length of the earlobe”, at least unilateral				
**Reference standard and target condition**	„A cardiac radiologist analysed the cineangiograms and classified them as abnormal if they showed (...) narrowing greater than 50%”				
**QUADAS-2 domain**		QUADAS-2 item	Description	Judgement	
**Patient selection**	Risk of bias	Consecutive or random sample enrolled?	„consecutive”	Yes	Low
		Case-control design avoided?	Cross-sectional design	Yes	
		Inappropriate exclusions avoided?	No exclusions from 340 consecutive patients	Yes	
	Concerns regarding applicability		„chest pain in 178, recurrent symptoms after a myocardial infarction in 115 and suspected valvular or primary myocardial disease in 47”		High

**Index test**	Risk of bias	Interpreted without knowledge of the results of the reference standard?	„without knowing the results of the coronary arteriography the examiner assessed the earlobe”	Yes	Low
		Pre-specified threshold?	Definition above	Yes	
	Concerns regarding applicability		In line with the review question		Low
**Reference standard**	Risk of bias	Likely to correctly classify the target condition?	„narrowing greater than 50%”	Yes	Low
		Interpreted without knowledge of the results of the index test?	Not stated	Unclear	
	Concerns regarding applicability		In line with the review question		Low
**Flow and timing**	Risk of bias	Appropriate interval between index test and reference standard?	Both examined during one visit	Yes	Low
		All patients received the same reference standard?	Coronary angiography performed on all 340 patients	Yes	
		All patients included in the analysis?	Results of all 340 patients presented	Yes	

**First author (year)**	**Salamati (2008) [38]**				
**Population**	„106 consecutive patients, referred from cardiologists for angiography, (...) in four academic hospitals in Teheran.”				
**Index test**	Positive if „clear cut diagonal crease reaching to both borders of the earlobe” or superficial or not extening all the way across the earlobe („according to Ishii classification”), at least unilateral				
**Reference standard and target condition**	„CAD was defined as the presence of angiographic coronary stenosis >50% of the luminal diameter in at least one of the three major epicardial arteries.”				
**QUADAS-2 domain**		QUADAS-2 item	Description	Judgement	
**Patient selection**	Risk of bias	Consecutive or random sample enrolled?	„consecutive”	Yes	Low
		Case-control design avoided?	„cross-sectional study”	Yes	
		Inappropriate exclusions avoided?	No exclusions from 106 consecutive patients	Yes	
	Concerns regarding applicability		Indications for invasive coronary angiography not stated who underwent planned procedure		Low

**Index test**	Risk of bias	Interpreted without knowledge of the results of the reference standard?	„assessed the earlobe (...) without knowing the results of the coronary angiographies"	Yes	Low
		Pre-specified threshold?	Definition above	Yes	
	Concerns regarding applicability		In line with the review question		Low
**Reference standard**	Risk of bias	Likely to correctly classify the target condition?	„angiographic coronary stenosis >50%"	Yes	Low
		Interpreted without knowledge of the results of the index test?	„The coronary cineangiograms were analyzed by (...) cardiologists unaware of the presence of the ELC."	Yes	
	Concerns regarding applicability		In line with the review question		Low
**Flow and timing**	Risk of bias	Appropriate interval between index test and reference standard?	Both examined during one visit	Yes	Low
		All patients received the same reference standard?	„coronary angiogram (...) performed in all participants."	Yes	
		All patients included in the analysis?	Results of all 106 patients presented	Yes	

**First author (year)**	**Shmilovich (2012) [29]**				
**Population**	„430 consecutive patients without a history of coronary artery intervention who underwent CT angiography"				
**Index test**	„(...) wrinkle-like line extending diagonally from the tragus across the lobule to the rear edge of the auricle of the ear...", present if bilateral				
**Reference standard and target condition**	„studied coronary arteries using CT angiography", „for CAD severity, we evaluated the presence of angiographically significant CAD (cutoff ≥50% stenosis)."				
**QUADAS-2 domain**		QUADAS-2 item	Description	Judgement	
**Patient selection**	Risk of bias	Consecutive or random sample enrolled?	„consecutive"	Yes	Low
		Case-control design avoided?	Cross-sectional design	Yes	
		Inappropriate exclusions avoided?	„excluding those with coronary artery bypass surgery (n=23), previous coronary artery stenting (n=2), or an uninterpretable scan (n=4)"	Yes	

	Concerns regarding applicability		„Indications for undergoing CT angiography were chest pain in 46%, previous equivocal results from other imaging modalities in 34%, screening before noncardiac operations in 11%, screening because of multiple and unbalanced coronary risk factors in 9%.”		Low
**Index test**	Risk of bias	Interpreted without knowledge of the results of the reference standard?	„Immediately before performance of CT angiography, patients were examined for DELC”	Yes	Low
		Pre-specified threshold?	Definition above	Yes	
	Concerns regarding applicability		In line with the review question		Low
**Reference standard**	Risk of bias	Likely to correctly classify the target condition?	„angiographically significant CAD (cutoff ≥50% stenosis).”	Yes	Low
		Interpreted without knowledge of the results of the index test?	„CT angiography interpretation was performed by (...) readers who were blinded to the presence or absence of DELC.”	Yes	
	Concerns regarding applicability		In line with the review question		Low
**Flow and timing**	Risk of bias	Appropriate interval between index test and reference standard?	Both examined during one visit	Yes	Low
		All patients received the same reference standard?	CTA performed on all 430 patients	Yes	
		All patients included in the analysis?	Results of all 430 patients presented	Yes	

**First author (year)**	**Toyosaki (1986) [45]**			
**Population**	„Coronary angiography was performed on 200 patients from", „Japanese adult patients (...) who were outpatients or inpatients at Jichi Medical School Hospital”			
**Index test**	„EC was considered to be present if there was a clear-cut crease which extended entirely across the earlobe of at least one ear”			
**Reference standard and target condition**	„angiographic evidence of coronary artery disease (greater than 50% luminal narrowing)”			
**QUADAS-2 domain**		QUADAS-2 item	Description	Judgement

**Patient selection**	Risk of bias	Consecutive or random sample enrolled?	Not stated	Unclear	Unclear
		Case-control design avoided?	Cross-sectional design	Yes	
		Inappropriate exclusions avoided?	Not stated	Unclear	
	Concerns regarding applicability		„patients with angina pectoris and/or previous myocardial infarction"		Low
**Index test**	Risk of bias	Interpreted without knowledge of the results of the reference standard?	Not stated	Unclear	Unclear
		Pre-specified threshold?	Definition above	Yes	
	Concerns regarding applicability		In line with the review question		Low
**Reference standard**	Risk of bias	Likely to correctly classify the target condition?	„angiographic evidence of coronary artery disease (greater than 50% luminal narrowing)"	Yes	Low
		Interpreted without knowledge of the results of the index test?	Not stated	Unclear	
	Concerns regarding applicability		In line with the review question		Low
**Flow and timing**	Risk of bias	Appropriate interval between index test and reference standard?	Both examined during one visit	Yes	Low
		All patients received the same reference standard?	Coronary angiography performed on all 200 patients	Yes	
		All patients included in the analysis?	Results of all 200 patients presented	Yes	

**First author (year)**	**Wang (2016) [42]**				
**Population**	„558 consecutive participants (...) who underwent coronary angiography for either suspected or known coronary atherosclerosis at the First Affiliated Hospital of Nanjing Medical University"				
**Index test**	Positive if „deep diagonal crease (>1 mm) extending obliquely from the tragus towards the outer border of the ear lobe and covering at least two-thirds of the length of the ear lobe", results for both unilateral and bilateral sign were presented				
**Reference standard and target condition**	„Significant CHD was defined as at least one major epicardial vessel with >50% stenosis, whereas the control was defined as <50% stenosis within each of the major epicardial vessels"				
**QUADAS-2 domain**		QUADAS-2 item	Description	Judgement	

**Patient selection**	Risk of bias	Consecutive or random sample enrolled?	„consecutive”	Yes	Low
		Case-control design avoided?	„cross-sectional study”	Yes	
		Inappropriate exclusions avoided?	„exclusion criteria were (..) spastic angina pectoris, heart failure, infectious process within 2 weeks of enrolment, adrenal dysfunction or thyroid dysfunction”	Yes	
	Concerns regarding applicability		Patients underwent procedure „for either suspected or known coronary atherosclerosis”		Low
**Index test**	Risk of bias	Interpreted without knowledge of the results of the reference standard?	„observers blinded to participants' diagnoses”	Yes	Low
		Pre-specified threshold?	Definition above	Yes	
	Concerns regarding applicability		In line with the review question		Low
**Reference standard**	Risk of bias	Likely to correctly classify the target condition?	„Significant CHD was defined as at least one major epicardial vessel with >50% stenosis”	Yes	Low
		Interpreted without knowledge of the results of the index test?	Not stated	Unclear	
	Concerns regarding applicability		In line with the review question		Low
**Flow and timing**	Risk of bias	Appropriate interval between index test and reference standard?	Both examined during one visit	Yes	Low
		All patients received the same reference standard?	Coronary angiography performed on all 588 patients	Yes	
		All patients included in the analysis?	Results of all 558 patients presented	Yes	

**First author (year)**	**Wu (2014) [43]**
**Population**	„449 consecutive Chinese", „were prospectively recruited from consecutive in-hospital cases that underwent CAG”
**Index test**	Positive if „deep diagonal crease (>1 mm) extending obliquely from the tragus towards the outer border of the ear lobe, covering at least two-thirds of the ear lobe length", at least unilateral

**Reference standard and target condition**	„CAD was defined by diameter stenosis of >50% in one or more of the epicardial arteries (...) revealed by CAG"				
**QUADAS-2 domain**		QUADAS-2 item	Description	Judgement	
**Patient selection**	Risk of bias	Consecutive or random sample enrolled?	„consecutive"	Yes	Low
		Case-control design avoided?	Cross-sectional design	Yes	
		Inappropriate exclusions avoided?	„Exclusion criteria included those who have an earring or piercing"	Yes	
	Concerns regarding applicability		Indications for coronary angiography not stated and we assume that substantial percent underwent planned procedure		Low
**Index test**	Risk of bias	Interpreted without knowledge of the results of the reference standard?	„observers who were blinded to patients' diagnosis"	Yes	Low
		Pre-specified threshold?	Definition above	Yes	
	Concerns regarding applicability		In line with the review question		Low
**Reference standard**	Risk of bias	Likely to correctly classify the target condition?	„CAD was defined by diameter stenosis of >50% in one or more of the epicardial arteries (...) revealed by CAG"	Yes	Low
		Interpreted without knowledge of the results of the index test?	Not stated	Unclear	
	Concerns regarding applicability		In line with the review question		Low
**Flow and timing**	Risk of bias	Appropriate interval between index test and reference standard?	Both examined during one visit	Yes	Low
		All patients received the same reference standard?	Coronary angiography performed on all 449 patients	Yes	
		All patients included in the analysis?	Results of all 449 patients presented	Yes	
