# Supplementary material for: Diagonal Earlobe Crease (Frank’s Sign) for Diagnosis of Coronary Artery Disease: A Systematic Review of Diagnostic Test Accuracy Studies
Source: J Clin Med. 2021 Jun 25;10(13):2799. doi: 10.3390/jcm10132799 (PMC8268092; doi:10.3390/jcm10132799)
Supplement: Supplementary file 1 [file jcm-10-02799-s001.zip › File S3.pdf]

List of 21 excluded studies at full-text assessment stage with reasons

Author	Reason of exclusion	Comment
Bawaskar [26]	Methodological and descriptive issues	888 outpatients presented between years 2012 and 2015 and complaining of chest pain were evaluated. Authors stated that "All the patients examined for presence of diagonal ear lobe crease (DELC) and investigated for BMI, height, electrocardiography changes, and coronary angiography", whereas in another paragraph there is mentioned that "About 177 patients undergone coronary angiography, of these, 22 had normal coronaries with no DELC. About 155 (87,5%) cases with DELC showed lesion in one vessel, 24 (15.49%); two vessels, 79 (50,96%); and three vessels, 52 (33.54%)." According to these results, sensitivity as well as specificity of diagonal earlobe crease in this study were 100%. Additionally, indications for invasive coronary angiography and threshold of positive examination were not defined and many contradictions were present in the manuscript. All these issues led us to decision of exclusion of this study.
Bernabo [33]	No access	Published in 1983, only basic bibliographic information available without abstract nor any contact details to authors
Blodgett [23]	Case-control design, not relevant reference standard	77 patients with history of myocardial infarction were compared with 77 age- and sex-matched controls. Moreover, information obtained from patients' medical records was reference standard and any additional diagnostic studies were not performed.
Dytfeld [18]	Not relevant reference standard	Reference standard was 70% or more stenosis of at least one epicardial coronary artery in invasive coronary angiography.
Elliott [20]	Not relevant reference standard	Reference standard was 75% or more stenosis of at least one epicardial coronary artery in invasive coronary angiography.
Evrengul [15]	Not relevant reference standard	Reference standard was 70% or more stenosis of at least one epicardial coronary artery in invasive coronary angiography.
Farrell [24]	Case-control design, not relevant reference standard	23 hospitalized patients with myocardial infarction were compared with age- and sex-matched control group of 23 surgical patients with no history of previous myocardial infarction, angina, or intermittent claudication.
Gral [22]	Not relevant target condition and reference standard	Target condition was defined as cardiovascular disease including coronary artery disease, cerebrovascular disease, arterial hypertension, and others. Moreover, information obtained from patients' medical records was reference standard and any additional diagnostic studies were not performed.
Haft [35]	No access	Published in 1979, only basic bibliographic information available without abstract nor any contact details to authors
Kaukola [9]	Case-control design	Kaukola et al. study consisted of two independent parts. In the first one, 219 hospitalized patients with myocardial infarction were compared with age- and sex-matched control group of 290 railway employees recruited out of hospital.
	Not relevant population	In the second part, authors evaluated 286 patients undergoing invasive coronary angiography of which 102 were consecutive subjects admitted to the hospital for coronary angiography or coronary by-pass surgery and another 184 were selected from patients who had undergone such procedure in the preceding years and were invited to take part in this study. As we stated in our protocol, we were interested in population of patients with suspected disease in which examination of diagonal earlobe crease may have the practical value.

Kuon [17]	Not relevant reference standard	Reference standard was 70% or more stenosis of at least one epicardial coronary artery in invasive coronary angiography.
Lesbre [16]	Not relevant reference standard	Reference standard was 75% or more stenosis of at least one epicardial coronary artery in invasive coronary angiography.
Lichstein [25]	Case-control design, not relevant reference standard	531 hospitalized patients with myocardial infarction were compared with control group of 305 patients with no clinical evidence of coronary artery disease (no angina pectoris, previous myocardial infarction, Q waves, ST segment, or T wave abnormalities in electrocardiography).
Lichstein [27]	Letter	
Mirić [32]	No access	Published in 1990, only basic bibliographic information and abstract available without any contact details to authors
Montesinos [28]	Letter	
Moraes [21]	Not relevant reference standard	247 patients admitted to “acute general hospital” were studied. Diagnosis of coronary heart disease was made basing on “angina, congestive heart failure or myocardial infarction and with reference to electrocardiograms and coronary angiograms”, however any other details of reference standard were not available. Contact information was limited to authors’ affiliation address.
Schreiber [31]	No access	Published in 1986, only basic bibliographic information available without abstract nor any contact details to authors
Shibuya [19]	Not relevant reference standard	Reference standard was 75% or more stenosis of at least one epicardial coronary artery in invasive coronary angiography.
Shmilovich [30]	Subgroup analysis	Subgroup analysis of patients with chest pain from the entire cohort evaluated in the previous study (indications for computed tomography angiography were chest pain, equivocal results of functional test, assessment prior to noncardiac surgery, and multiple cardiovascular risk factors) which met inclusion criteria and was enrolled in our systematic review.
Wermut [34]	No access	Published in 1980, only basic bibliographic information available without abstract nor any contact details to authors

Numbering of references are in line with the main body of the article.
